# A novel circRNA, circNUP98, a potential biomarker, acted as an oncogene via the miR‐567/ PRDX3 axis in renal cell carcinoma

**DOI:** 10.1111/jcmm.15629

**Published:** 2020-07-30

**Authors:** Rui Yu, Jie Yao, Yu Ren

**Affiliations:** ^1^ Zhejiang Key Laboratory of Pathophysiology Department of Biochemistry and Molecular Biology Medical School Ningbo University Ningbo China; ^2^ School of Marine Science Ningbo University Ningbo China; ^3^ Department of Urologic Surgery Ningbo Urology and Nephrology Hospital, Ningbo Yinzhou NO2. Hospital Ningbo China

**Keywords:** circNUP98, miR‐567, PRDX3, renal cell carcinoma

## Abstract

In recent years, plenty of studies found that circular RNAs (circRNAs) were essential players in the initiation and progression of various cancers including the renal cell carcinoma (RCC). However, the knowledge about the circRNAs in carcinogenesis is still limited. Dysregulated expression of circNUP98 in RCC tissues was identified by the circular RNA microarray. RT‐PCR was performed to measure the expression of circNUP98 in 78 pairs of RCC tissues and adjacent normal tissues. Survival analysis was conducted to explore the association between the expression of circNUP98 and the prognosis of RCC. The function and underlying mechanisms of circSMC3 in RCC cells were investigated by RNAi, CCK‐8, Western blotting, bioinformatic analysis, ChIP assay, circRIP assay and dual luciferase reporter assay. CircNUP98 was up‐regulated in both RCC tissues and cell lines, and high expression of circNUP98 was correlated with poor prognosis of RCC patients. Silencing of circSMC3 inhibited the proliferation and promoted the apoptosis in a caspase‐dependent manner in RCC cells. Mechanistically, we revealed that silencing of circ NUP98 inhibited RCC progression by down‐regulating of PRDX3 via up‐regulation of miR‐567. Furthermore, STAT3 was identified as an inducer of circ NUP98 in RCC cells. CircNUP98 acts as an oncogene by a novel STAT3/circ NUP98/miR‐567/PRDX3 axis, which may provide a potential biomarker and therapeutic target for the treatment of RCC.

## 1 INTRODUCTION

Renal cell carcinoma (RCC) is a common urological system solid tumour, with high incidence and accounts for about 1.8% of cancer‐related death worldwide.^1^ There are three subtypes of RCC known as clear cell renal cell carcinoma (ccRCC) (75%‐80%), papillary RCC (10%‐15%) and chromophobe RCC (5%‐10%).^2^ Although great progress has been made in the detection and management of RCC, the overall survival (OS) of RCC patients still remains dismal. Therefore, it is necessary to explore the molecular mechanisms underlying progression of RCC and to identify effective diagnostic biomarker or therapeutic target for RCC.

Recently, it was found that non‐coding RNAs (ncRNAs) played an essential in the initiation and progression of various human cancers including the RCC. Circular RNAs (circRNAs) are a group of novel ncRNAs derived from exon regions of coding genes.^3^ Besides the canonical splicing mode of linear RNAs, circRNAs can be formed by back‐splicing events among which an upstream splice acceptor site joins with a downstream splice donor site resulting in exon circularization.^4^ CircRNAs are lack of 5’ (cap) and 3’ (polyadenylation) ends and can be linked by a covalent bond to form a closed‐loop structure.^5^ This unique structure makes circRNAs less vulnerable to degradation by exonuclease RNase R.^6^ Due to their stability and abundance in body fluid, different circRNAs have been identified as potential biomarkers for the diagnosis and prognosis of various cancers including the RCC.^7^ Mechanistically, circRNAs functioned as sponges of miRNAs and thereby protecting the downstream mRNAs from degradation mediated by miRNAs.^8^

miRNAs are another type of ncRNA and act mainly via negatively regulation of the target genes at post‐transcription level.^9^ Amounting evidence suggested that miRNAs were involved in the tumorigenesis and progression of RCC.^10^ For instance, it was found that miR‐935 promoted the migration and invasion of RCC cells via regulation of IREB2.^11^ In contrast, miR‐765 functioned as a tumour suppressor and inhibited formation of lipids in RCC through inhibition of PLP2.^12^ Despite the increasing investigations regarding circRNAs, there is still relatively little known about the roles of cirRNAs in RCC.

In the current study, we found a novel circRNA, circNUP98, that was significantly up‐regulated in RCC tissues and cells. In addition, circNUP98 was closely correlated with poor prognosis and tumour grade in RCC patients. Functional studies showed that down‐regulation of circNUP98 inhibited progression of RCC both in vitro and in vivo. Furthermore, mechanistic investigation reveals that circNUP98 is under the regulation of STAT3 and exerts its effects via the miR‐567/PRDX3 axis in RCC cells.

## 2 MATERIALS AND METHODS

### 2.1 Human RCC specimens

Seventy‐eight pairs of RCC tissues and adjacent normal tissues were obtained from RCC patients from Ningbo Yinzhou No. 2 Hospital from August 2013 to September 2015. None of the patients received any treatment before surgery. All patients signed informed consent forms before inclusion in this study. The tissues were collected instantly after surgical resection and stored in liquid nitrogen. All specimens were confirmed by two experienced physicians. Tumour staging and grading were confirmed according to the AJCC TNM 2010 classification system and WHO/ISUP 2004 system, respectively. This study was approved by Ethics Committee of Ningbo Yinzhou No. 2 Hospital. Additionally, a cohort of 65 RCC patients with clinicopathological parameters was followed up. The follow‐up time ranged from 1 to 60 months. The follow‐up interval began on the date of surgery and ended on the date of disease progression.

### 2.2 Cell culture

Human renal normal cells (293K) and RCC cells (ACHN, 786‐O, Caki‐1) were all obtained from the Shanghai Bank of Cell Culture, Chinese Academy of Sciences. Cells were maintained in RPMI1640 medium (Invitrogen) supplemented with 10% foetal bovine serum (FBS, Invitrogen), 100 U/mL penicillin (Sigma) and 100 μg/mL streptomycin (Sigma). The cells were maintained in humidity air at 37℃ with 5% CO_2_, and the culture medium was changed every 2 days.

### 2.3 Cell transfection

For knockdown of circNUP98, short hairpin RNAs (shRNAs) against circNUP98, PRDX3, STAT3 or the negative control were ordered from GenePharma and cloned into pLKO.1 TRC vector (Addgene, No. 10878). Lentivirus production and infection were performed according to the manufacturer's guide. For overexpression of PRDX3, STAT3, full‐length cDNA of PRDX3 and STAT3 were ordered and subcloned into pcDNA3.1 vector by GeneScript. Transfections were performed with 100 ng plasmids and Lipofectamine 2000 (Life Technologies). Before transfection, cells were seeded into 6‐well plates at a density of 2 × 10^5^ cells/well and cultured to around 70% confluence.

### 2.4 Cell viability assay

Cell viability was measured by the CCK‐8 assay kit (Beyotime) according to the manufacturer's guide. Briefly, cells were seeded into 96‐well plates at a density of 5 × 10^3^ cells/well and cultured in fresh medium mixed with CCK‐8 at a ratio of 10:1 for 2 hours. Absorbance at 450 nm of each well was measured using a microplate reader (BioTek).

### 2.5 Colony formation assay

After transfection for 24 hours, the cells were seeded into 6‐well plate at the density of 500 cells/well. Then, the cells were cultured in incubator and the medium was changed every 3 days in a total of 3‐week period. Then, the cells were fixed with paraformaldehyde (Sigma) and stained with crystal violet (Beyotime). The colony formation numbers were observed and counted under inverted light microscope (Olympus IX90).

### 2.6 Microarray analysis

Microarray analysis of expression of circRNAs was conducted using Arraystar Human circRNA Array V2.0. Total RNA was purified from 3 pairs of RCC and adjacent normal tissues. Microarray hybridization was conducted according to the standard protocols from Arraystar. In short, total RNA was treated with RNase R (Sigma) to remove the linear RNA, and then, the circRNAs were enriched. Then, RNAs were amplified for cRNA and labelled using the Arraystar Super RNA labelling kit (Arraystar). Then, these labelled RNAs were hybridized and the hybridization was scanned by the Scanner G2505C (Agilent).

### 2.7 RNA purification and RT‐PCR

Total RNA was extracted using TRIzol reagent (Beyotime) according to the company's protocol. The quantity and quality of RNA was measured using NanoDrop ND 2000 Spectrophotometer (Thermo Scientific Inc). Total RNA was reversely transcribed into cDNA using PrimeScript RT Reagent (TakaRa) according to the manufacturer's protocol. The expression levels of circSMC3, miR‐942 and other mRNAs were evaluated using the SYBR Premix Ex Taq (Takara). GAPDH and U6 were used as internal controls. The reaction conditions were as follows: 95°C for 10 minutes for 1 cycle, denaturation at 95°C for 30 seconds, annealing at 56°C for 1 minutes, extension at 72°C for 30 seconds for a total of 40 cycles. The relative gene expression was calculated using the 2^−ΔΔCt^ method, and the samples were run in triplicate.

### 2.8 Cellular apoptosis assay

The apoptosis of cells was assayed by the Annexin V‐fluorescein isothiocyanate/propidium iodide (FITC/PI) detection kit (Invitrogen) according to the manufacturer's instruction. Briefly, cells were seeded on the 6‐well plate at the density of 1 × 10^6^ cells/well. 24 hours later, cells were transfected and cultured for another 24 hours and then were collected by centrifugation at 500 g at room temperature. The supernatant was discarded, and pellets were washed three times with PBS. Subsequently, cells were rinsed with binding buffer and stained with 5 μL Annexin V‐FITC and 10 μL PI solution at room temperature for 30 minutes in the dark. Then, the status of apoptotic cells was determined by flow cytometry (FACSCalibur, BD Biosciences).

### 2.9 Migration and invasion assay

Migration of cells was measured by the wound healing assay.^13^ Cells were seeded into 6‐well plate at the density of 2 × 10^5^ cells/well and cultured to around 70% confluence. The monolayer was gently and slowly scratched with a sterile 20‐μL pipette tip and then was washed with PBS to remove the debris. Subsequently, the cells were incubated for another 24 hours and cell migration was photographed using an inverted light microscope (Olympus IX90). Invasion of cells was assayed using the transwell assay. In this assay, 1 × 10^5^ transfected cells were suspended in 200 μL of serum‐free medium and seeded into the top chambers of transwell (8 μm pore size, Corning) coated with Matrigel (BD Bioscience). The bottom chamber was filled with full medium as attractant. After incubation for 24 hours for invasion, non‐invaded cells were gently removed and cells invaded were fixed with 4% paraformaldehyde (Sigma), stained with crystal violet solution (Beyotime) for 30 minutes and visualized under a microscope (Olympus IX90) at × 100 magnification. All cells were counted in five randomly chosen microscopic fields.

### 2.10 Caspase‐3 activity assay

Caspase‐3 activity was measured using a caspase‐3 colorimetric assay kit (Abcam). Cells were seeded in the 24‐well plate at the density of 1 × 10^5^ cells/well. Then, cells were transfected for 24 hours and lysed in the provided lysis buffer and centrifuged at 10 000 × *g* for 1 minutes, and the supernatants were collected. Subsequently, equal amounts of protein were incubated with the substrate Z‐DEVD‐AMC at 37°C for 1 hours. The activity of caspase‐3 was determined at 405 nm using the microplate reader (Biotek). All experiments were performed at least three times.

### 2.11 Subcellular fraction assay

The location of circNUP98 was evaluated by using the PARIS^TM^ kit (Invitrogen) according to the company's guide. Briefly, cells were suspended in cytoplasm lysis buffer and centrifuged at 1500 rpm for 5 minutes. The cytoplasmic supernatant was collected and the pellet was re‐suspended in nucleus lysis buffer at 4°C for 1 hours, following centrifugation at 1500 rpm for 10 minutes. The RNAs derived from cytoplasmic and nuclear extracts were purified by TRIzol (Beyotime) according to the manufacturers guide. The expression levels of GAPDH (cytoplasm control), U6 (nucleus control) and circNUP98 in nucleus and cytoplasm were assayed by qRT‐PCR as described above.

### 2.12 ChIP assay

ChIP assay was performed using the MagnaChIP Kit (Millipore) according to the manufacturer's guide. The antibodies against STAT3 and IgG used in the ChIP assay were obtained from the Sigma. After incubation with beads provided by the kit, the precipitates were assayed by RT‐qPCR.

### 2.13 circRIP assay

circRIP assay was performed using the protocol from GeneSeed. Briefly, cells were sonicated after fixation with formaldehyde (Sigma). Then, the supernatant was incubated with the biotinylated circNUP98 or control probe (RioBio) and the magnetic streptavidin Dynabeads (Sigma). After total RNA extraction, the enrichment was measured by qRT‐PCR.

### 2.14 Luciferase activity assay

Dual luciferase reporter assays were performed using the co‐transfection of recombinant luciferase reporter vectors and indicated transfection plasmids into RCC cells. The wild‐type (wt) or mutated (mut) miR‐567 interacting sites in circNUP98 or PRDX3 sequence were used for constructing the pmirGLO‐circNUP98/PRDX3‐wt/mut. Besides, the pGL3‐circNUP98 promoter‐wt/Mut#1/2/3/4 reporter vectors were generated to measure the STAT3 binding ability to circNUP98 promoter. The mutations were constructed using the QuickChange^TM^ II Site‐Directed Mutagenesis kit (Stratagene) according to the manufacturer's protocol. Luciferase activity was monitored after 48 hours by Dual Luciferase Reporter Assay System (Promega).

### 2.15 Western blotting assay

Cells were lysed using the RIPA lysis buffer (Beyotime). The concentration of protein was calculated by BCA protein assay kit (Beyotime), and 20 μg of total protein was separated by 12% SDS‐PAGE and transferred onto PVDF membrane (Millipore). The membranes were blocked with skimmed milk for 1 hours at room temperature, and then, membrane was incubated with primary antibody overnight at 4°C. After that, the membrane was washed three times with PBS and incubated with corresponding HRP‐conjugated secondary antibody at room temperature for 1 hours. The membrane was visualized using ECL Prime Western Blotting Kit (Beyotime). All the primary and secondary antibodies were purchased from CST (Cellular Signaling Technology).

### 2.16 Statistically analysis

Statistical analyses were performed with SPSS 12.0 (IBM). Data are expressed as the mean ± SD. A one‐way ANOVA was used to determine the statistical difference between multiple groups. A post hoc test was used to calculate the statistical difference between two groups. *P* value < .05 (two‐tailed) was considered statistically significant.

## 3 RESULTS

### 3.1 A novel circRNA, circNUP98, was up‐regulated in RCC tissues and correlated with poor prognosis

**Figure 1:**
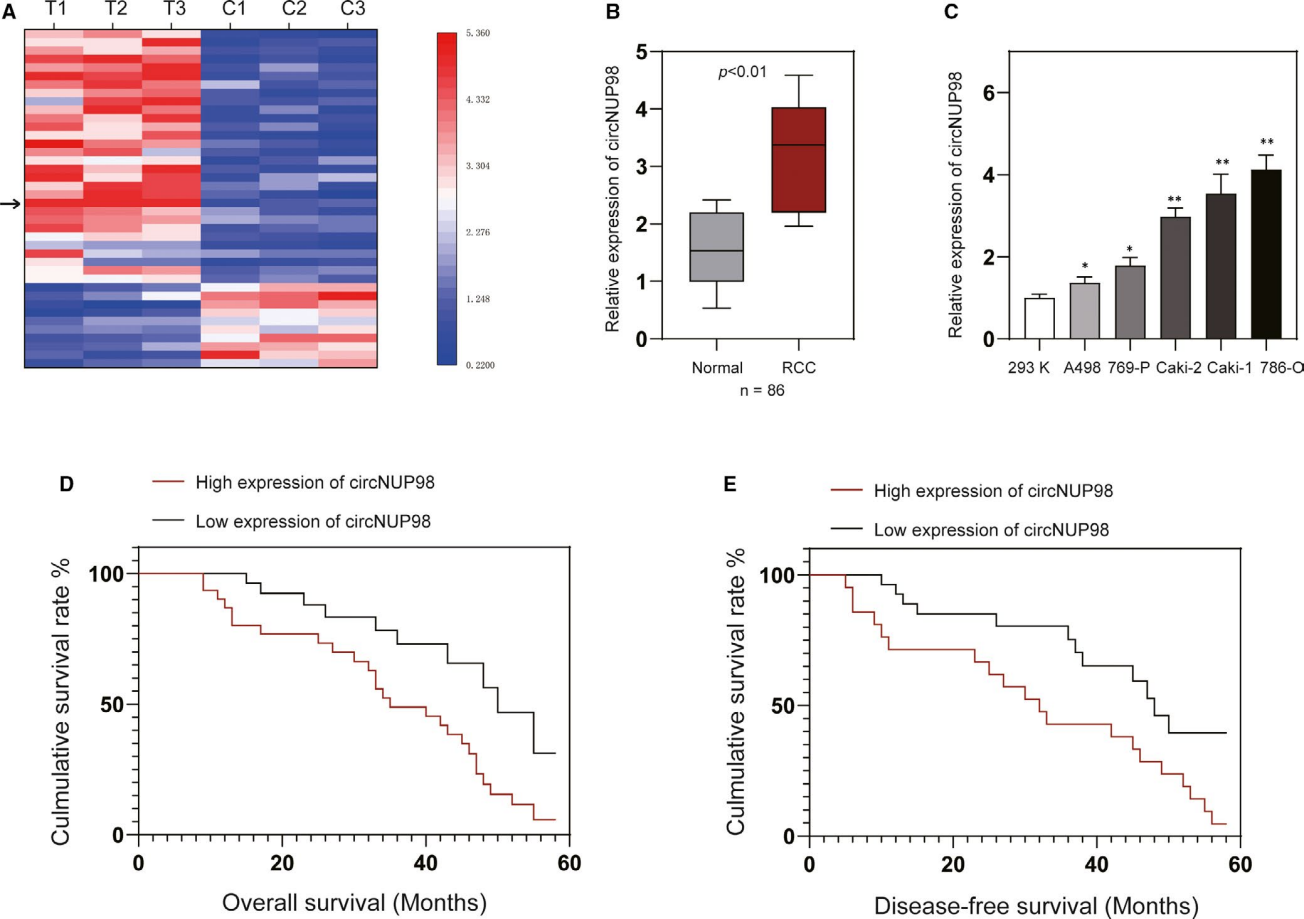
Expression of circNUP98 was increased in RCC tissues and correlated with prognosis of RCC patients. A, Heat map showing the circRNA expression profiles of RCC tissues and adjacent normal tissues. Arrow indicates the circNUP98. B, The levels of circNUP98 were measured by RT‐PCR in 78 pairs of RCC tissues and adjacent normal tissues. C, The expression of circSMC3 in human normal renal cells (293K) and RCC cells (A498, 767P, Caki‐1, Caki‐2, 786‐O). D, Overall survival and (E) disease‐free survival of RCC patients with high or low expression of circNUP98. Data were presented as mean ± SD. Experiments were performed at least three times. *P < .05; **P < .01; ***P < .001

Firstly, we applied circRNA microarray to analyse the expression profile of circRNAs in 3 pairs of RCC tissues and their adjacent normal tissues. Heat map showed up‐regulated and down‐regulated circRNAs, and hsa_circRNA_0000274 was the top up‐regulated one in RCC tissues (Figure 1A). We termed hsa_circRNA_0000274 as 'circNUP98' as it was derived from the *NUP98* gene according to the human reference genome. Next, we assayed the levels of circNUP98 in 78 pairs of RCC tissues and their adjacent normal tissues. It was found that the expression of circNUP98 was significantly increased in RCC tissues when compared to the adjacent normal tissues (Figure 1B). The expression levels of circNUP98 were also higher in RCC cells than normal renal cells (Figure 1C). Because the expression of circNUP98 in 786‐O and Caki‐1 cells was higher than other cells, we chose these two cell lines for the following investigations. Then, we divided the RCC patients into two groups (high, low) according to the median ratio of circNUP98 expression. Kaplan‐Meier analysis and log‐rank test indicated that RCC patients with high expression of circNUP98 had a poorer overall (Figure 1D) and disease‐free (Figure 1E) survival when compared with RCC patients with low expression of circNUP98. Moreover, analysis of the expression of circNUP98 and clinicopathological features of RCC patients showed that high expression of circNUP98 was correlated with advanced tumour stage (Table 1). Those data indicated that circNUP98 might act as an oncogene and correlate with poor prognosis of RCC patients.

**Table 1:** Relationship between circNUP98 expression and clinicopathological features of RCC patients

Characteristics	Cases (n = 78)	Expression of circSMC3		*P*‐value
		Low (n = 39)	High (n = 39)	
Gender				
Male	45	20	25	.252
Female	33	19	14	
Age (years)				
<60	46	24	22	.654
≥60	32	15	17	
Lymph node metastasis				
Yes	34	18	16	.648
No	44	21	23	
Distant metastasis				
Yes	32	13	19	.167
No	46	26	20	
Tumour size (cm)				
<5	45	25	20	.252
≥5	33	14	19	
TNM stage				
I + II	47	28	19	.037
III + IV	31	11	20	
Fuhrman				
I + II	38	21	17	.365
III + IV	40	18	22	

### 3.2 Silencing of circNUP98 affected the proliferation, migration, invasion and apoptosis of RCC cells

**Figure 2:**
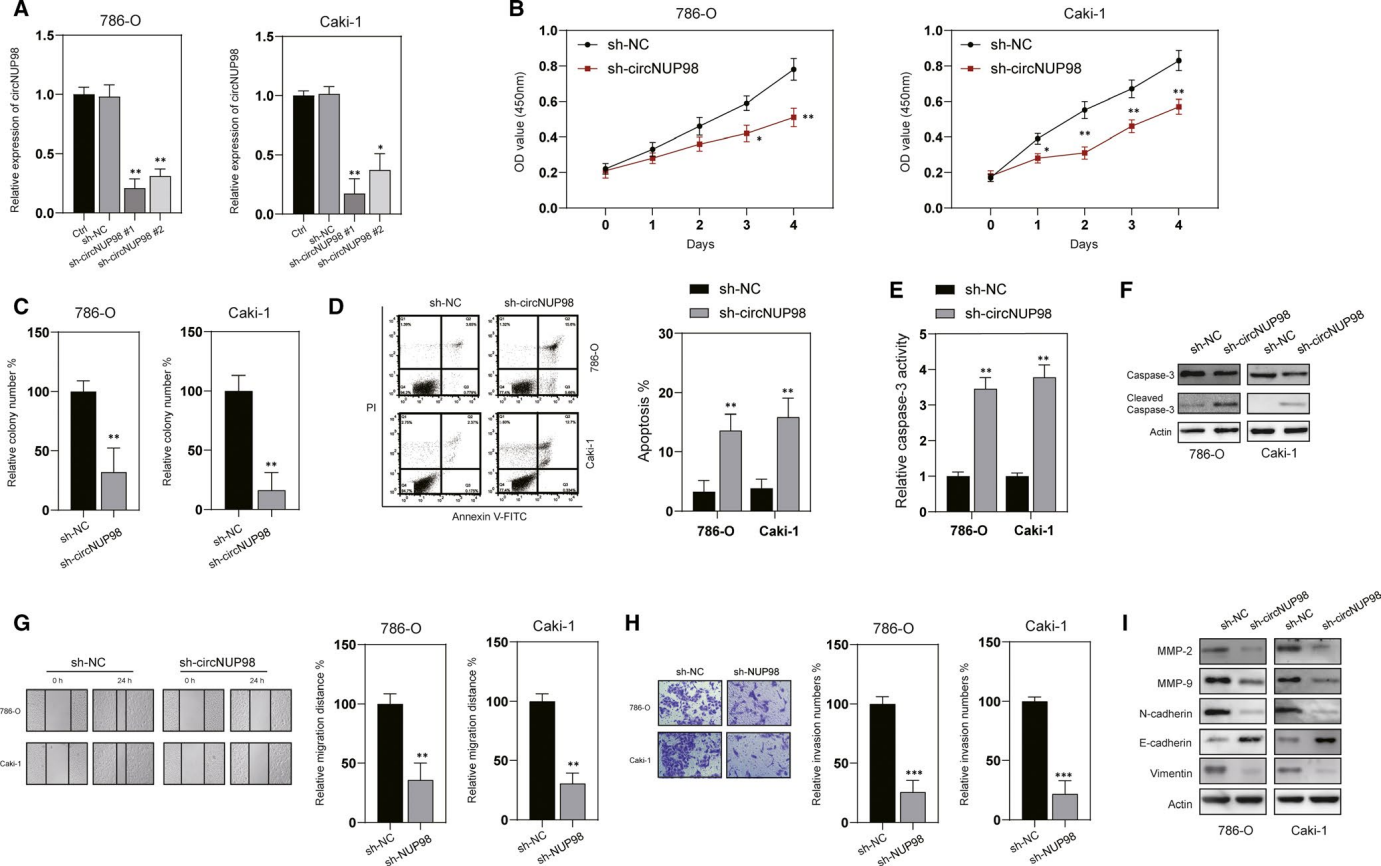
circNUP98 affects cell growth, migration, invasion and apoptosis of RCC cells. A, RCC cells were transfected as indicated, and the expression of circNUP98 was measured by RT‐PCR. B, Cell proliferation analysed by CCK‐8 assay after knockdown of circNUP98 by shRNA for indicated time in RCC cells. C, Colony formation assay was conducted after silencing of circNUP98 in RCC cells. D, Analysis of apoptosis by flow cytometry after silencing of circNUP98 in RCC cells for 48 h. E, Caspase‐3 activity was assayed after silencing of circNUP98 in RCC cells for 24 h. F, Western blot of caspase‐3 after down‐regulation of circNUP98 in RCC cells. G, Wound healing assay was performed after silencing of circNUP98 in RCC cells. H, Tranwell assay was performed after silencing of circNUP98 in RCC cells. I, The indicated proteins were assayed by western blotting after silencing of circNUP98. Data were presented as mean ± SD. Experiments were performed at least three times. *P < .05; **P < .01; ***P < .001

To explore the role of circNUP98 in RCC, we used two short hairpin RNAs (shRNAs) to target it. It was revealed that both of them significantly decreased the expression of circNUP98 (Figure 2A) without any effects on the linear NUP98 mRNA and protein levels (Data not shown) in RCC cells. As shRNA against circNUP98#1 (sh‐circNUP98#1) was more efficient, so it was chosen for the following experiments. CCK‐8 assay showed that the proliferation and colony formation of RCC cells were significantly inhibited after knockdown of circNUP98 (Figure 2B,C). Meanwhile, annexin V/PI assay showed that down‐regulation of circNUP98 triggered apoptosis of RCC cells (Figure 2D). Caspase‐3 activity assay and Western blotting also confirmed activation of caspase‐3 after silencing of circNUP98 in RCC cells (Figure 2E,F). Wound healing assay and Matrigel assay revealed that down‐regulation of circNUP98 repressed the migration and invasion of RCC cells (Figure 2G,H). Moreover, silencing of circNUP98 also inhibited the levels of MMP‐2/9 and markers of EMT process (Figure 2I). In short, all of these studies indicate that silencing of circNUP98 can inhibit the proliferation, migration and invasion and promote the apoptosis of RCC cells.

### 3.3 CircNUP98 acts as a sponge for miR‐567

**Figure 3:**
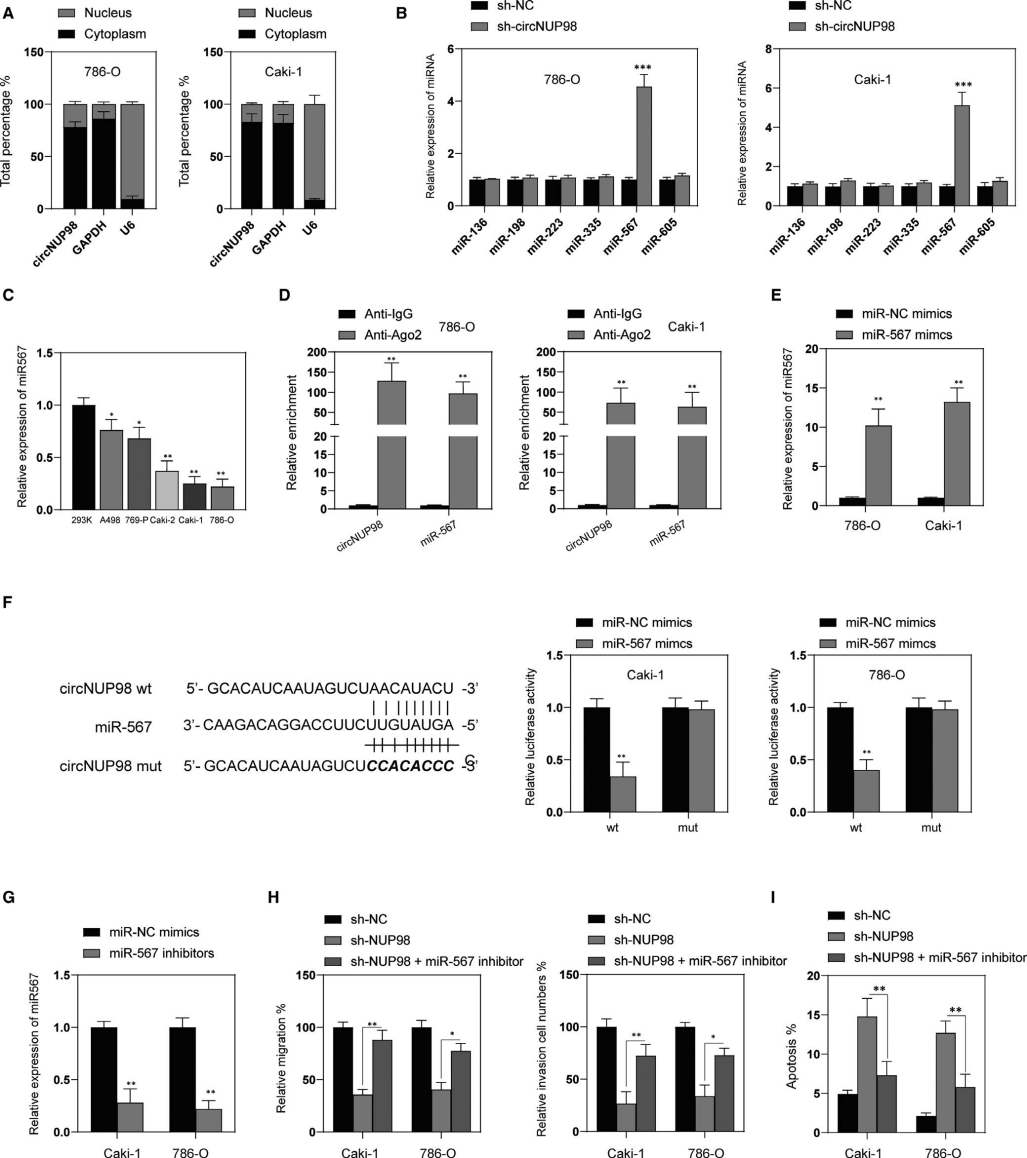
circNUP98 functions a sponge of miR‐567. A, Subcellular fraction analysis of localization of circNUP96, GAPDH mRNA and U6 mRNA. B, The levels of different miRNAs were measured by RT‐PCR after silencing of circNUP98 in RCC cells. C, The expression of miR‐567 in human normal renal cells (293K) and RCC cell lines (A498, 769‐P, Caki‐2, Caki‐1 and 786‐O) were measured by RT‐PCR. D, The enrichment of circNUP98 and miR‐567 was measured by RIP assay in RCC cells. E, RCC cells were transfected miR‐567 or miR‐NC mimics for 24 h, and then, the levels of miR‐567 were measured by RT‐PCR. F, The predicted binding sites between miR‐567 and circNUP98 (left) and relative luciferase activity in RCC cells co‐transfected with wt or mut luciferase reporters and miR‐567 mimics or corresponding negative control. G, RCC cells were transfected with miR‐567 or miR‐NC inhibitors for 24 h, and then, the expression of miR‐567 was measured by RT‐PCR. H, RCC cells were transfected as indicated for 24 h, and the migration (left) and invasion (right) were measured by wound healing and Matrigel assay, respectively. I, RCC cells were transfected as indicated for 48 h, and cellular apoptosis was analysed. Data were presented as mean ± SD. Experiments were performed at least three times. *P < .05; **P < .01; ***P < .001

We investigated the subcellular localization of circNUP98 by subcellular fraction assay in order to further elucidate the specific mechanism of circNUP98 involved in RCC. It was demonstrated that circNUP98 is mainly located in the cytoplasm of RCC cells, indicating the post‐transcriptional regulatory mechanism of circNUP98 (Figure 3A). Hence, we investigated whether circNUP98 exerted its function as a miRNA sponge in RCC cells. By bioinformatical tools (StarBase 2.0, TargetScan), five candidate miRNAs with the binding potential with circNUP98 were chosen. RT‐PCR showed that only miR‐568 was remarkably up‐regulated after silencing of circNUP98 in RCC cells (Figure 3B). It was also found that expression of miR‐567 was decreased in RCC cells compared with normal renal cells (Figure 3C). Furthermore, RIP assay showed a remarked enrichment of circNUP98 and miR‐567 was detected in anti‐Ago2 group (Figure 3D). To further investigate the interaction between circNUP98 and miR‐567, we transfected RCC cells with miR‐567 mimics, which can significantly up‐regulate the expression of miR‐567 in RCC cells (Figure 3E). Bioinformatic analysis revealed the binding sites between miR‐567 and circNUP98, and dual luciferase activity assay was performed to verify it (Figure 3F, left). It was found that the luciferase activity of pmirGLO‐circNUP98‐wt was significantly reduced, whereas no changes of the luciferase activity of pmirGLO‐circNUP98‐mut were observed (Figure 3F, right). Further, rescued experiments were conducted after the expression of miR‐567 was inhibited by miR‐567 inhibitor in RCC cells (Figure 3G). As indicated in Figure 3H,I, inhibition of miR‐567 could reverse the effect of silencing of circNUP98 on migration, invasion and apoptosis of RCC cells. In brief, circNUP98 interacts with miR‐567 in RCC cells.

### 3.4 PRDX3 is a direct target of miR‐567 in RCC cells

**Figure 4:**
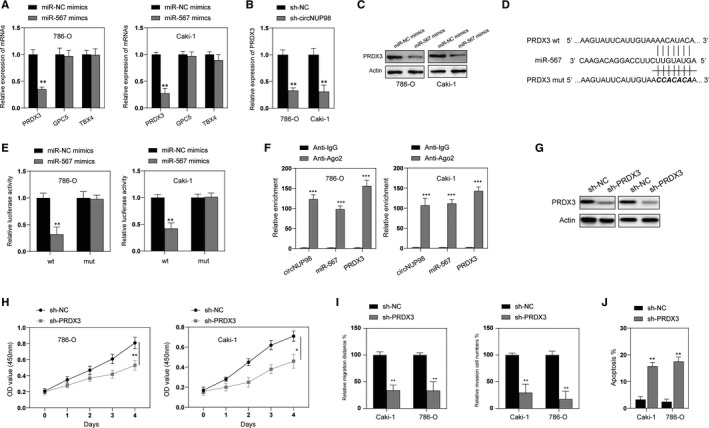
PRDX3 is a target of miR‐567. A, RCC cells were transfected with miR‐NC or miR‐567 mimics for 24h, and mRNA levels of PRDX3, GPC5 and TBX4 were measured by RT‐PCR. B, RCC cells were transfected with sh‐NC or sh‐circNUP98 for 24 h, and the expression of circNUP98 was assayed by RT‐PCR. C, RCC cells were transfected with miR‐NC or miR‐567 mimics for 24 h, and the protein levels of PRDX3 were measured by Western blotting. D, The predicted binding sites between miR‐567 and PRDX3 3’UTR region. E, relative luciferase activity in RCC cells co‐transfected with wt or mut luciferase reporters and miR‐567 mimics or corresponding negative control. F, The enrichment of circNUP98, miR‐567 and PRDX3 mRNA was measured by RIP assay in RCC cells. G, RCC cells were transfected with sh‐NC or sh‐PRDX3 for 24 h, and the protein levels of PRDX3 were measured by Western blotting. H, After silencing of PRDX3, the proliferation of RCC cells was measured by CCK‐8 assay at indicated time‐point. I, After silencing of PRDX3 for 24 h, the migration and invasion of RCC cells were measured by wound healing and Matrigel assay, respectively. J, After silencing of PRDX3 for 24 h, the apoptosis of RCC cells was measured by flow cytometry. Data were presented as mean ± SD. Experiments were performed at least three times. *P < .05; **P < .01; ***P < .001

Next, we used online bioinformatical tools (StarBase 2.0, PicTar) to predict the downstream target gene of miR‐567 in RCC cells. After overexpression of miR‐567, PRDX3 expression was significantly inhibited in RCC cells (Figure 4A). Meanwhile, silencing of circNUP98 also down‐regulated the expression of PRDX3 (Figure 4B) and miR‐567 could reduce protein levels of PRDX3 in RCC cells (Figure 4C). Thus, we chosen PRDX3 to continue this investigation. The binding site between PRDX3 and miR‐567 was predicated by the bioinformatical tools (Figure 4D). Dual luciferase activity assay showed that the luciferase activity of pmirGLO‐PRDX3‐wt was markedly enhanced, whereas the luciferase activity of pmirGLO‐PRDX3‐mut showed no significant changes among different transfected cells (Figure 4E). Additionally, RIP assay showed that RNAs (circNUP98, miR‐567 and PRDX3) were all enriched in anti‐Ago2 but not anti‐IgG group (Figure 4F). In order to further examine the role of PRDX3, we used shRNA to successfully inhibit the expression of PRDX3 in RCC cells (Figure 4G). CCK‐8 assay showed that silencing of PRDX3 significantly inhibited the proliferation of RCC cells (Figure 4H). Meanwhile, silencing of PRDX3 also inhibited the migration, invasion and promoted apoptosis of RCC cells (Figure 4I,J). Taken together, those data suggest that PRDX3, which acts as an oncogene in RCC cells, is a direct target of miR‐567 in RCC cells.

### 3.5 Overexpression of PRDX3 reversed the effects of silencing of circNUP98 in RCC cells

**Figure 5:**
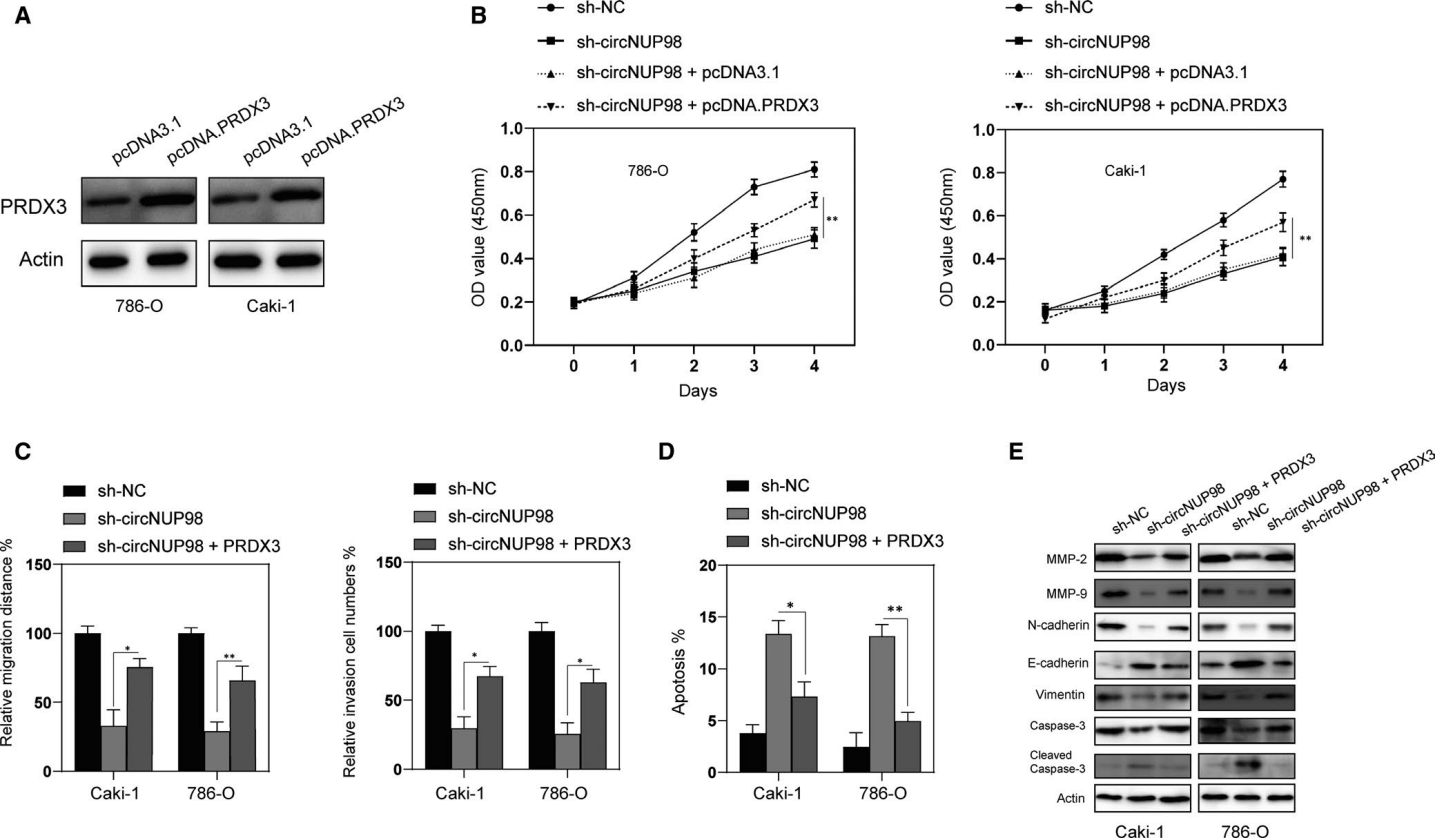
Overexpression of PRDX3 reversed the effects of silencing of circNUP98. A, RCC cells were transfected with pcDNA3.1 or pcDNA.PRDX3 for 24 h, and the protein levels of PRDX3 were measured by Western blotting. B, RCC cells were transfected as indicated, and the proliferation of cells was measured by CCK‐8 assay at indicated time‐points. C, RCC cells were transfected as indicated, and migration and invasion of cells were measured by wound healing assay and Matrigel assay, respectively. D, RCC cells were transfected as indicated, and cellular apoptosis was measured by flow cytometry. E, RCC cells were transfected as indicated, and cellular lysates were subjected to Western blotting with indicated antibodies. Data were presented as mean ± SD. Experiments were performed at least three times. *P < .05; **P < .01; ***P < .001

Furthermore, rescue experiments were performed to testify the role of circNUP98/miR‐567/PRDX3 axis in regulating the tumorigenesis of RCC. Firstly, we transfected RCC with pcDNA.PRDX3 to up‐regulate the expression of PRDX3 in RCC cells (Figure 5A). As indicated in Figure 5B, the inhibitory effects of silencing of circNUP98 on proliferation could be reversed by overexpression of PRDX3 in RCC cells. Migration and invasion ability impaired by silencing of circNUP98 was promoted by forced expression of PRDX3 in RCC cells (Figure 5C). Furthermore, apoptosis triggered by silencing of circNUP98 could also be blocked by overexpression of PRDX3 in RCC cells (Figure 5D). In addition, effects of silencing of circNUP98 on MMPs, EMT markers and caspase‐3 also be reversed by overexpression of PRDX3. Taken together, those data indicate that circNUP98 hindered progression of RCC via the miR‐567/PRDX3 axis.

### 3.6 STAT3 promotes circNUP98 expression in RCC cells

**Figure 6:**
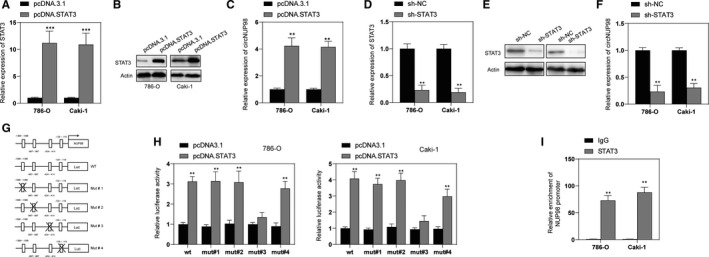
STAT3 induces the expression of circNUP98 in RCC cells. A, RCC cells were transfected with pcDNA3.1 or pcDNA.STAT3 for 24 h, and the mRNA levels of STAT3 were measured by RT‐PCR. B, RCC cells were transfected with pcDNA3.1 or pcDNA.STAT3 for 24 h, and the protein levels of STAT3 were measured by Western blotting. C, RCC cells were transfected with pcDNA3.1 or pcDNA.STAT3 for 24 h, and the expression of circNUP98 was measured by RT‐PCR. D, RCC cells were transfected with sh‐NC or sh‐STAT3 for 24 h, and the mRNA levels of STAT3 were measured by RT‐PCR. E, RCC cells were transfected with p sh‐NC or sh‐STAT3 for 24 h, and the protein levels of STAT3 were measured by Western blotting. F, RCC cells were transfected with sh‐NC or sh‐STAT3 for 24 h, and the expression of circNUP98 was measured by RT‐PCR. G, The predictive promoter region of NUP98, top; the reporter constructs of wild‐type Luc and its mutated derivatives are also shown in bottom. H, The interaction between STAT3 and NUP98 promoter was validated through luciferase reporter assay. I, The binding ability between STAT3 and NUP98 promoter was testified by ChIP assay in RCC cells. Data were presented as mean ± SD. Experiments were performed at least three times. **P < .01; ***P < .001

Furthermore, we also tried to unveil the upstream mechanism of circNUP98. By using online bioinformatic tools (http://genome.ucsc.edu/; http://bioinfo.life.hust.edu.cn/hTFtarget#!/), STAT3 was predicted to bind the promoter region of *NUP98*. To test this prediction, we transfected RCC cells with pcDNA.STAT3 that successfully enhanced the expression of STAT3 (Figure 6A,B). RT‐PCR was performed, and it was revealed that overexpressing of STAT3 promoted expression of circNUP98 in RCC cells (Figure 6C). Then, we silenced the expression of STAT3 in RCC cells by sh‐STAT3 (Figure 6D,E). It was found that the expression of circNUP98 was inhibited after silencing of STAT3 (Figure 6F). In order to further elucidate the relationship between STAT3 and circNUP98, online bioinformatic tools (JASPAR and MatInspector) were used and then potential binding sites between STAT3 and *NUP98* promoter were predicted (Figure 6G, top). Serial mutants of the *NUP98* gene promoter were created based on the location of the STAT3‐binding sites to unveil the transcriptional regulatory sites (Figure 6G, bottom). Luciferase reporter assay showed that overexpression of STAT3 increased the luciferase activity of pGL3‐NUP98 promoter‐wt/mut #1/mut #2 but not mut #3 in RCC cells (Figure 6H). These data suggested that the promoter region of *NUP98* between −424 and −414 was responsible for STAT3 mediated activation. Furthermore, ChIP assay also indicated that STAT3 could bind with promoter region of *NUP98* (Figure 6I). Taken together, these data indicated that STAT3 activates transcription of *NUP98* and may thereby increase the expression of circNUP98 in RCC cells.

### 3.7 Silencing of NUP98 enhanced the chemosensitivity of RCC cells and inhibited the progression of RCC in a xenograft mouse model

**Figure 7:**
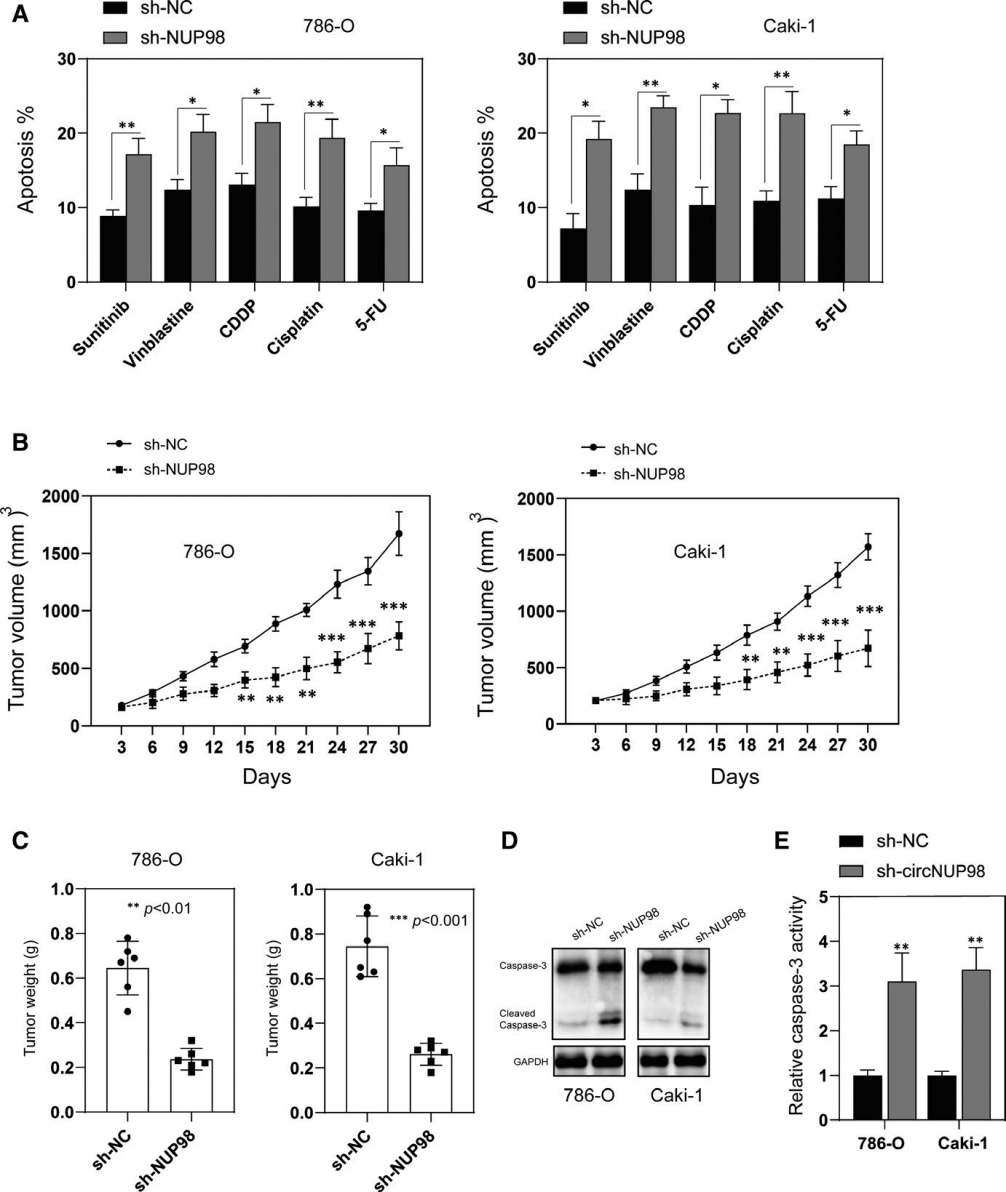
Silencing of circNUP98 increased chemosensitivity of RCC cells and inhibited the growth of RCC in vivo. A, RCC cells were transfected with sh‐NC or sh‐circNUP98 for 24 h, and then, the cells were exposure to different chemotherapy agents for another 24 h, and cellular apoptosis was analysed by flow cytometry. B, RCC cells stably transfected with sh‐NC or sh‐circNUP98 and inoculated into nude mice, and tumour volumes were measured as different time‐points. C, Mice were killed 30 days after inoculation, and the tumour weight was measured. D, Xenografts were subjected to Western blotting analysis of caspase‐3. E, Xenografts were subjected to caspase‐3 activity assay. Data were presented as mean ± SD. Experiments were performed at least three times. *P < .05; **P < .01; ***P < .001

Next, we evaluated the effects of down‐regulation on the chemosensitivity of RCC cells. Interestingly, it was found that silencing of circNUP98 increased the chemosensitivity of RCC cells to various anti‐tumour agents (Sunitinib 10 μmol/L, Vinblastine 1 μmol/L, CDDP 1 μmol/L, Cisplatin 10 μmol/L, 5‐FU 20 μmol/L) (Figure 7A). Finally, the effects of circNUP98 on the progression of RCC cells were evaluated in a nude mice xenograft model. 786‐O and Caki‐1 cells transfected with recombinant lentiviral vector containing sh‐circNUP98 or sh‐NC were inoculated into the nude mice. It was found that sh‐circNUP98 significantly inhibited the growth of tumour in vivo, as tumour size and weight were both significantly decreased when compared with the controls (Figure 7B,C). Moreover, xenograft tumour tissues were subjected to Western blotting and caspase‐3 activity assay. It was found the activation of caspase‐3 was greatly enhanced after silencing of circNUP98 (Figure 7D,E). Those data indicated that down‐regulation of circNUP98 not only enhanced the chemosensitivity of RCC cells but also inhibited the growth of RCC cells in vivo.

## 4 DISCUSSION

In recent years, circRNAs became one of the research hotspots due to its essential roles in various biological activities.^14^ circRNAs can be applied as potential cancer biomarkers and therapeutic targets because of their stability and abundance.^15^ In the present study, we firstly reported that circNUP98 was up‐regulated in human RCC tissues and cell lines compared with adjacent normal tissues and normal renal cells. In addition, high expression of circNUP98 was significantly correlated with tumour stage and poor survival of RCC patients. Therefore, circNUP98 could be a promising independent prognostic biomarker for RCC. We also investigated in effects of circNUP98 on RCC progression and elucidated the underlying mechanisms. We revealed that silencing of circNUP98 inhibited the proliferation, migration and invasion of RCC cells but promoted the apoptosis of RCC cells.

CircRNAs have been found played different roles in various cancers including the RCC. For instance, circRAPGEF5 is down‐regulated in RCC tissues and it inhibits the growth and metastasis of RCC via regulation of miR‐27a/TXNIP axis.^16^ In contrast, circPCNXL2 promotes the proliferation and invasion of RCC cells via sponging of miR‐153 and thereby further regulates the expression of ZEB2.^17^ To date, there is little knowledge about the function of circNUP98 in RCC. However, *NUP98*, where is circNUP98 derived from, acted as an oncogene in haematological cancers and breast cancer.^18^, ^19^ Our findings suggest that NUP98 might also exert oncogenic effects in RCC and it would be interesting to test it.

It was well recognized that circRNAs comport gene expression regulatory functions via regulation of the expression of miRNAs.^20^ miR‐567 has been reported to be significantly down‐regulated in breast cancer tissues, and up‐regulation of miR‐567 markedly inhibited the proliferation and migration of breast cancer cells in vitro.^21^ In addition, miR‐567 inhibited the proliferation, migration and invasion of osteosarcoma cells via targeting FGF5.^22^ One of the most important findings in our study was that knockdown of circNUP98 increased the expression of miR‐567 in RCC cells. Moreover, inhibition of miR‐567 significantly abrogated the influences of silencing of circNUP98 on tumorigenesis of RCC cells. In line with previous studies, our data also indicated that miR‐567 might be a tumour suppressor in RCC cells. Noteworthy, a recent study reported that miR‐567 could be sponged by circRNA cMars and inhibition of miR‐567 contributed to the progression of lung adenocarcinoma.^23^ This discrepancy might be caused by different tumour tissues, thereby more investigations regarding the function of miR‐567 are necessary.

PRDX3, belongs to the peroxiredoxins (PRDXs) family, is mainly located on the mitochondrial and serves as an important antioxidant protein that serves as the target for about 90% H_2_O_2_ produced in the matrix.^24^ PRDX3 has been found associated with metastasis and poor survival in uveal melanoma.^25^ Knockdown of PRDX3 inhibited the growth of hepatocellular carcinoma cells.^26^ In accordance with those studies, we found that silencing of RPDX3 repressed the migration, invasion and promoted the apoptosis of RCC cells. Our study suggested that PRDX3 might also be used as a potential target for the treatment of RCC and further study is required to test this.

What's more, we also showed that circNUP98 was under the regulation of STAT3. STAT3 has been recognized as a transcription factor and oncogene in various cancers including the RCC.^27^ Activation of STAT3 alone is sufficient to induce cell transformation, showing a strong oncogenic potential, and promotes the initiation and progression of RCC.^28^ Our findings provide novel insights into the function of STAT3 and the regulatory mechanism upstream of circRNA.

In summary, we found that circNUP98 was significantly up‐regulated, whereas miR‐567 was decreased in human RCC. Mechanisms of investigations unveiled important roles of circNUP98 in regulating the progression of RCC. We also found that circNUP98 functioned as a sponge of miR‐567 and, in turn, promoted the expression of PRDX3. Moreover, circNUP98 was under the regulation of STAT3. Our findings revealed a novel axis of STAT3/circNUP98/miR‐567/PRDX3 that could be used as a potential target for RCC.

## CONFLICT OF INTEREST

The authors confirm that there are no conflict of interest.

## AUTHOR CONTRIBUTIONS

**Rui Yu:** Conceptualization (equal); Data curation (equal); Formal analysis (lead); Investigation (lead); Methodology (lead); Project administration (lead); Resources (lead); Software (lead); Supervision (equal); Validation (equal); Visualization (equal); Writing‐original draft (lead); Writing‐review & editing (lead). **Jie Yao:** Conceptualization (supporting); Data curation (supporting); Formal analysis (supporting); Investigation (equal); Methodology (supporting); Project administration (equal); Resources (equal); Software (equal); Validation (supporting); Visualization (supporting); Writing‐original draft (supporting); Writing‐review & editing (supporting). **Yu Ren:** Conceptualization (supporting); Data curation (supporting); Formal analysis (supporting); Investigation (supporting); Methodology (equal); Project administration (equal); Resources (equal); Software (equal); Supervision (lead); Validation (equal); Visualization (equal); Writing‐original draft (equal); Writing‐review & editing (equal).

## Data Availability

Data are available upon reasonable request.
